# Validation of the Fibromyalgia Survey Questionnaire within a Cross-Sectional Survey

**DOI:** 10.1371/journal.pone.0037504

**Published:** 2012-05-25

**Authors:** Winfried Häuser, Eva Jung, Brigitte Erbslöh-Möller, Mechthild Gesmann, Hedi Kühn-Becker, Franz Petermann, Jost Langhorst, Thomas Weiss, Andreas Winkelmann, Frederick Wolfe

**Affiliations:** 1 Department Internal Medicine I, Klinikum Saarbrücken, Saarbrücken, Germany; 2 Department of Psychosomatic Medicine and Psychotherapy, Technische Universität München, München, Germany; 3 Rheumatology Medical Practice, Neunkirchen, Germany; 4 Psychosomatic Medicine and Pain Therapy Medical Practice, Herford, Germany; 5 Psychosomatic Medicine and Pain Therapy Medical Practice, Zweibrücken, Germany; 6 Centre of Clinical Psychology and Rehabilitation, Universität Bremen, Bremen, Germany; 7 Department Internal Medicine V (Intergrative Medicine), Kliniken Essen-Mitte, Essen, Germany; 8 Outpatient Clinic Mannheim, Mannheim, Germany; 9 Department of Physical Medicine and Rehabilitation, Klinikum der Universität München, München, Germany; 10 National Data Bank for Rheumatic Diseases, Wichita, Kansas, United States of America; 11 University of Kansas School of Medicine, Wichita, Kansas, United States of America; Tehran University of Medical Sciences, Iran (Islamic Republic of)

## Abstract

The Fibromyalgia Survey Questionnaire (FSQ) assesses the key symptoms of fibromyalgia syndrome. The FSQ can be administrated in survey research and settings where the use of interviews to evaluate the number of pain sites and extent of somatic symptom intensity and tender point examination would be difficult. We validated the FSQ in a cross-sectional survey with FMS patients. In a cross-sectional survey, participants with physician diagnosis of FMS were recruited by FMS-self help organisations and nine clinical institutions of different levels of care. Participants answered the FSQ (composed by the Widespread Pain Index [WPI] and the Somatic Severity Score [SSS]) assessing the Fibromyalgia Survey Diagnostic Criteria (FSDC) and the Patient Health Questionnaire PHQ 4. American College of Rheumatology 1990 classification criteria were assessed in a subgroup of participants. 1,651 persons diagnosed with FMS were included into analysis. The acceptance of the FSQ-items ranged between 78.9 to 98.1% completed items. The internal consistency of the items of the SSS ranged between 0.75–0.82. 85.5% of the study participants met the FSDC. The concordance rate of the FSDC and ACR 1990 criteria was 72.7% in a subsample of 128 patients. The Pearson correlation of the SSS with the PHQ 4 depression score was 0.52 (p<0.0001) and with the PHQ anxiety score was 0.51 (p<0.0001) (convergent validity). 64/202 (31.7%) of the participants not meeting the FSDC criteria and 152/1283 (11.8%) of the participants meeting the FSDC criteria reported an improvement (slightly too very much better) in their health status since FMS-diagnosis (Chi^2^ = 55, p<0.0001) (discriminant validity). The study demonstrated the feasibility of the FSQ in a cross-sectional survey with FMS-patients. The reliability, convergent and discriminant validity of the FSQ were good. Further validation studies of the FSQ in clinical and general population settings are necessary.

## Introduction

The publication of American College of Rheumatology (ACR) preliminary diagnostic criteria for fibromyalgia syndrome (FDC) [1] eliminated the tender point examination required for the clinical diagnosis of FMS by the ACR 1990 classification criteria [2]. Because most of the ACR 2010 items can be obtained by self-administration, the FDC were slightly modified so that complete self-administration would be possible by the Fibromyalgia Survey Diagnostic Criteria (FSDC). The FSDC were developed in a longitudinal study of patients of the National Data Bank for Rheumatic Diseases by substituting a count of three symptoms for the physician's (0–3) evaluation of the extent of somatic symptom intensity by a questionnaire assessing the number of pain sites and somatic symptom severity. Patients who satisfy FSDC meet the following 3 conditions: 1) Widespread Pain Index (WPI) ≥7/19 pain sites and Symptom Severity Score (SSS) ≥5/12 or WPI between 3–6/19 and SSS ≥9/12; 2) Symptoms have been present at a similar level for at least 3 months; 3) The patient does not have another disorder that would otherwise sufficiently explain the pain [3]. The conditions 1 and 2 can be assessed by the Fibromyalgia survey questionnaire (FSQ) including the WPI and SSS. The sum of the WPI and the SSS constitutes the Fibromyalgianess Scale (FS) or polysymptomatic distress scale as a measure of physical and psychological symptom intensity (distress) which can be applied to every disease. The FS can be used to track disease status. The assessment of the key symptoms of FMS by the FSQ allows administration in survey research and settings where the use of interviews to evaluate the number of pain sites and extent of somatic symptom intensity would be difficult.

In this study we provided the first translation of the FSQ into German language and validated the FSQ for the first time in a cross-sectional survey with FMS-patients in Germany.

## Methods

### Clinical institutions

Participants of the study were recruited by the two largest German FMS-self help organisations and nine clinical institutions. The specialties of the clinical institutions were pain medicine and psychotherapy (N = 3), rheumatology (N = 2), complementary and alternative medicine (N = 2), physical therapy (N = 1) and pain therapy (N = 1). The settings were outpatient (N = 6), inpatient (N = 2) and day clinic (N = 1). The levels of care were secondary (N = 6) and tertiary care (N = 1) and rehabilitation (N = 1).

From November 1, 2010 to April 30, 2011 all consecutive patients with an established or first diagnosis of FMS of the participating study centres were asked by the physicians of these centres to take part in the study. All participating physicians had more than 10 years exprerience in the management of FMS-patients. The questionnaires were handed out by the physicians of the centres with a standardized letter explaining the focus of the study. The questionnaires were returned by the patients in a closed and anonymous envelope and kept away from the charts. In 4 centres a tender point examination was performed according to a standardised protocol [4].

### Self-help organisations

The package of questionnaires was sent by the central office of the German League for people with Arthritis and Rheumatism to their regional offices with the request that the leaders of the local self-help groups distribute the FSQ during the meetings to the group members (FMS-patients). Group members were asked to fill out the questionnaires separately outside the group meetings and not to discuss it with other group members.

The German Fibromyalgia Association included the package in the issue 4/2010 of its member journal “Optimist” dispatched by post to all members.

The questionnaires were returned by the patients by post to the central office. Moreover, the questionnaires were available on the homepages of both self-help organisations. After downloading and completing they could be sent by mail, fax or email to the central offices. Employees of both central offices removed the personal identifying information and sent the questionnaires to the coordinating study centre.

### Inclusion- and exclusion criteria

Members of the self-help organisations should report that the diagnosis of FMS had been established by a physician. Participants without (reported) physician diagnosis of FMS were excluded.

The patients of the study centres should have been previously or currently diagnosed with FMS according to the ACR 1990 classification criteria [3] or the Association of the Medical Scientific Societies in Germany (AWMF) criteria [5]. In four study centres the ACR 1990 criteria (2) were reevaluated during study examination. A diagnostic work-up including a complete physical examination and defined laboratory tests according to the German guideline on the management of FMS were performed in every patient of the study centres in the past or during the study [6]. Patients with somatic diseases sufficiently explaining the pain sites of the WPI (e.g. highly active inflammatory rheumatic disease) and patients who were not able to read German were excluded.

### Questionnaires

Demographic data (age, sex, family status, educational level, current professional status, member of a FMS-self help organisation) and medical data (Years since chronic widespread pain and FMS-diagnosis] were assessed by a questionnaire used in a previous multicenter FMS – study [5].

Patients were asked how their health status has changed over the years since the diagnosis of FMS according to their opinion (1 = very much worse, 2 = much worse, 3 = slightly worse, 4 = no change, 5 = slightly better, 6 = much better, 7 = very much better).

The FSQ included the Symptom Severity Score (SSS) with 3 major symptoms (fatigue, trouble thinking or remembering, waking up tired [unrefreshed]) which can be coded 0–3 (0 = not present to 3 = extreme) and three additional symptoms (Pain or cramps in lower abdomen, depression, headache), which can be coded to be present (1) or not present (0) (total suscore 0–3). These three items are surrogates for somatic symptom burden item of the ACR 2010 criteria (reference). The SSS ranges from 0–12. The Widespread Pain Index (WPI) includes 19 non-articular pain sites [2] (see table 1). The English version of the SSS had been forward and backtranslated by four German physicians, two of whom had worked for several years in the USA. We used the validated German version of the WPI [7].

**Table 1:** Fibromyalgia survey questionnaire.

I. Using the following scale, indicate for each item the level of severity over the past week by checking the appropriate box.		
0: No problem		
1: Slight or mild problems; generally mild or intermittent		
2: Moderate; considerable problems; often present and/or at a moderate level		
3. Severe: continuous, life-disturbing problems		
Fatigue	□ 0 □ 1 □ 2 □ 3	
Trouble thinking or remembering	□ 0 □ 1 □ 2 □ 3	
Waking up tired (unrefreshed)	□ 0 □ 1 □ 2 □ 3	
II. During the past 6 months have you had any of the following symptoms?		
Pain or cramps in lower abdomen	□ Yes □ No	
Depression	□ Yes □ No	
Headache	□ Yes □ No	
**III. Joint/body pain**		
Please indicate below if you have had pain or tenderness over the past 7 days in each of the areas listed below. Please make an X in the box if you have had pain or tenderness. Be sure to mark both right side and left side separately		
□ Shoulder, left	□ Upper leg, left	□ Lower back
□ Shoulder, right	□ Upper leg, right	□ Upper back
□ Hip, left	□ Lower leg, left	□ Neck
□ Hip, right	□ Lower leg, right	
□ Upper arm, left	□ Jaw, left	□ No pain in any of these areas
□ Upper arm, right	□ Jaw, right	
□ Lower arm, left	□ Chest	
□ Lower arm, right	□ Abdomen	
IV. Overall, were the symptoms listed in I–III above generally present for at **least 3 months**?	□ Yes □ No	

The 4-item Patient Health Questionnaire-4 (PHQ-4) is an ultra-brief self-report questionnaire that consists of a 2-item depression scale (PHQ-2) and a 2-item anxiety scale (GAD-2). A score of 3-or-greater on the depression subscale represents a reasonable cut-point for identifying potential cases of major depression or other depressive disorder, a score of 3-or-greater on the anxiety subscale represents a reasonable cut-point for generalized anxiety, panic, social anxiety, and posttraumatic stress disorders. The PHQ 4 total score can serve as measure for psychological distress [8]. We used the validated German version of the PHQ 4 [9].

### Validation methods and hypotheses

The methods used to validate the FSQ were as follows:

1. Patient acceptability (acceptance) of the FSQ was assessed by the proportion of missing or invalid items. The proportion of missing or invalid items should be approximately equal to those in surveys of German patients with chronic liver diseases [10] and celiac disease [11].
2. The reliability of the SSS was assessed by internal consistency (Cronbach's α coefficient) which measures the overall correlation between items within a scale. A level of 0.7 and higher is considered desirable [12].
3. Face (content) validity was assessed by the think aloud technique [13] of five phycians (pain medicine, psychosomatic medicine, rheumatology) and five FMS-patients of local self-help groups not participating in the study who verbalized their thoughts processes while filling out the FSQ.
4. Convergent validity of the SSS and FS was determined by the Pearson correlation with the total sum score of the PHQ 4. The convergent validity is fulfilled when the scale scores for related concepts show moderate to high correlation (correlation coefficient 0.4 to 0.8) [12].
  Convergent validity of the FSDC was determined by comparing the concordance rates of self-reported diagnosis of FMS made by a physician (members of self-help organisations) with the FSDC and of physician - established diagnosis of FMS (participants of clinical centres) with FSDC. Based on previous studies of the concordance rates of different FMS-diagnostic criteria [5], [14] we expected concordance rates between 70–80%.
5. Discriminant validity was tested by the following hypothesis: Longitudinal studies demonstrated that persons diagnosed with FMS can switch between criteria positive and criteria negative states [15]. Therefore we assumed, that patients who will not meet the FSDC at the time of evaluation, will report more frequently that their health status has improved since the diagnosis of FMS.

### Statistical analysis

The data were entered by four pairs of study assistants into a preconstructed excel-data sheet. The entering of data was checked by two authors at random and on plausibility during descriptive data analysis. Missing items of the SSS, WPI and PHQ 4 were coded as zero. Patients were excluded from analysis if all items of SSS and/or WPI and/or PHQ4 were not answered.

### Support

The participants of the study did not receive any reimbursement. Material costs were covered by the participating institutions.

### Ethics

The requirements of data protection and medical professional secrecy were respected by all study investigators. All participants gave their informed written consent to the study. The study had been specifically aproved by the ethical committee of the Ludwig Maximilian Universität München and by the review boards of all study centers.

## Results

### Study participants

There were no data available concerning how many patients contacted by the self-help organisation did not meet the inclusion criteria or refused to take part in the study. The German League for people with Arthritis and Rheumatism estimated that approximately 10 000 of their members were FMS-patients. The German Fibromyalgia Association is reported to have approximately 4000 members with FMS.

123 patients of the clinical samples did not meet the primary inclusion criteria and 40 of contacted patients refused to take part in the study. 1694 persons returned the questionnaires, of which 1143 (69.2%) had been contacted via self-help organisations. 43 of 1694 contacted persons were excluded due to total missing items in the WPI (N = 40) or SSS (N = 3). The questionnaires of at least 10 persons who were excluded due to missing WPI-items did not include the WPI due to an organisational mistake. 1651 persons were included into analysis.

The total study sample was composed of mainly middle-aged women with a long duration of CWP and FMS-diagnosis. In 30 patients, FMS was diagnosed recently for the first time (see table 2). 881/1633 (54.6%) participants scored > = 3 on the PHQ 4 depression scale and 889/1633 (54.4%) scored > = 3 on the PHQ 4 anxiety scale.

**Table 2:** Demographic and clinical characteristics of the total study sample.

Variable	N Total	Mean (SD), lowest and highest value	N (%)
Sex female	1647		1562 (94.8)
Age	1644	54.1 (9.8), 19–84	
Living with partner/in family	1641		1562 (77.4)
Educational level	1640		
No school finished			27 (1.7)
Primary school			558 (34.0)
Secondary school			676 (41.2)
High school			131 (8.0)
University			248 (15.1)
Current professional status	1637		
School			10 (0.6)
Working without sick leave			529 (31.1)
Working, sick leave			287 (14.9)
Applying for early retirement due to FMS			151 (8.9)
Without job			21 (1.2)
Homemaker			186 (10.9)
Pensioner			604 (35.6)
Member of a FMS self-help organisation	1640		994 (60.6)
Years since chronic widespread pain	1629	16.4 (11.6), 0.25–61	
Years since FMS-diagnosis	1596	6.5 (5.5), 0–41	
Somatic severity score (0–12)	1653	8.3 (2.6), 0–12	
Widespread pain index (0–19)	1653	11.6 (3.2), 0–19	
Fibromyalgianess Total score (0–31)	1650	19.9 (5.5); 5–31	
Patient Health Questionnaire 4 Total score (0–12)	1652	6.0 (0.1), 0–12	

### Validation

#### Acceptance

The participants included into analysis completed the SSS and PHQ 4 items as follows: Fatigue 1620 (98.1%), trouble thinking 1609 (97.4%), waking up tired 1618 (98.0%), pain in lower abdomen 1449 (87.8%), depression 1589 (96.2%), headache 1303 (78.9%), three months duration of all symptoms 1375 (83.3%), loss of interest 1622 (98.2%), feeling down 1623 (98.3%), nervousness 1632 (98.8%), worries 1612 (97.6%).

#### Reliability (Internal consistency)

Cronbach's alpha of the SSS was 0.65 and of the FS was 0.71.

#### Face validity

Two patients felt insecure where to indicate pain in the elbows and knees in the WPI because these pain sites were not mentioned. Two physicians felt puzzled by the different time frames of the FSQ. One physician wondered why abdominal pain was assessed both in the SSS and in the WPI.

#### Convergent validity

The Pearson correlation of the SSS with the PHQ 4 total score was 0.56 (p<0.0001) and of the FS with PHQ4 total score was 0.48 (p<0.001).

1411 (85.5%) participants of the total sample met the FSDC of FMS. 1351 (95.7%) reported a WPI ≥7 and a SSS ≥5 and 60 (4.4%) reported a WPI 3–6 and a SSS ≥9.

The diagnosis of FMS according to the ACR 1990 criteria was reevaluated at the date of appointment in 128 patients in 4 study centres with previously or actually diagnosed FMS. The mean of TPC was 13.8 (SD 3.5) (range 0–18). 107/128 (83.6%) participants met the ACR 1990 classification criteria of FMS. The concordance rate of the FSDC and ACR 1990 criteria was 72.7% (see table 3).

**Table 3:** Concordance rate of American College (ACR) 1990 classification criteria and Fibromyalgia Survey Diagnostic criteria (FSDC) (128 patients of four clinical centres).

ACR 1990 and FDSC positive	Only ACR 1990 positive	Only FDSC positive	Neither ACR 1990 and FDSC positive	Concordance rate ACR 1990 and FDSC
88 (68.8%)	15 (11.7%)	20 (15.6%)	5 (3.9%)	72.7%

#### Discriminant validity

64/202 (31.7%) of the participants not meeting the FSDC criteria and 152/1283 (11.8%) of the participants meeting the FSDC criteria reported an improvement (slightly too very much better) of their health status since FMS-diagnosis (Chi^2^ = 55, p<0.0001).

## Discussion

### Summary of main findings

In this study we provided the first translation of the FSQ into another language and validated the FSQ for the first time in a cross-sectional survey with 1651 FMS-patients. The acceptability, reliability and validity of the FSQ met the predefined quality criteria pointed out in the validation hypotheses.

### Relation to other studies

The proportion of missing items in the SSS ranged from 1.9 to 21.1%. The item with 21% missing was the headache item. We cannot explain the low completion rate of this item. The range was higher than the one of a disease specific health-related questionnaire in a survey with 522 patients with celiac disease which was 0.2–11.2 [11] and with 202 chronic liver patients was 0.4–2.8% [10].

The overall concordance rate of physician made diagnosis of FMS and FSDC was 85.5%. In a subsample the concordance of FSDC with ACR 1990 criteria was 72.7%. These concordance rates were similar with the ones of a study in an US rheumatologic practice in which the concordance rate of the ACR 1990 and clinical criteria of FMS was 72% [14].

The study results highlight the problem of defining cut-off values for continuous symptom disorders such as FMS. 14.5% of the patients who had once been diagnosed with FMS did not meet the FSDC criteria of FMS at the time of the survey. Most notably, 32% of these patients reported an improvement of health status since FMS-diagnosis. In a longitudinal study with 1,555 fibromyalgia patients meeting the FSDC citeria at study entry conversion from and to criteria positive status was common during 7,448 semi-annual observations for up to 11 years. During follow-up 716 patients (44.0%) failed to meet criteria at least once, and at study closure 24.3% failed to meet criteria [1]. In the long-term management of these patients the assessment of the amount of distress (“fibromyalgianess” or polysymptomatic distress) is possible by summing the SSS and the WPI [3].

The study confirms the high levels of distress reported by FMS patients and the concepualisation of FMS a continuum disorder which can be located at the extreme end of the continuum of distress [16], [17].

### Limitations

The study did not include FMS-patients from primary care settings.

There is no gold standard how to deal with missing values. We decided not to use imputation methods because the percentage of missing values was one criterion of validation.

Testing of the test-retest reliability and responsiveness to change was not possible within a cross-sectional survey. The test-retest reliability of a WPI ≥7 in FMS-patients of different clinical settings was 100% and the intraclass correlation of the WPI was 0.78 over a period of 4–12 weeks [7].

Because there is no gold standard of the clinical diagnosis of FMS [18], the assessment of the criterion validity of the FSDC were not possible. We used the ACR 1990 criteria as a common standard for the assessment of convergent validity. Due to the study design it was not possible to perform a TPC in all patients.

### Conclusions

#### Research

The study demonstrated the feasibility, reliability and validity of the FSQ in a survey with German FMS-patients. Further validation studies of the FSQ in other countries and/or languages are necessary. A standardization of the different time frames of the questions of the FSQ should be considered.

#### Clinical practice

The FSDC are not be used for self-diagnosis or as substitute for a physician's diagnosis. The FSQ can be used to gather information about the key symptoms of FMS and the extent of somatic symptom reporting, but the interpretation and assessment of questionnaire validity belongs to the physician [19].
